# Regional neuroinflammation induced by peripheral infection contributes to fatigue-like symptoms: a [^18^F]DPA-714 positron emission tomography study in rats

**DOI:** 10.3389/fimmu.2023.1261256

**Published:** 2023-11-09

**Authors:** Danxi Li, Di Hu, Yuta Ochi, Wakiko Arakaki, Aya Mawatari, Mika Shigeta, Yuping Wu, Emi Hayashinaka, Hiroyuki Neyama, Tsuyoshi Tahara, Yasuhiro Wada, Feng Li, Hisashi Doi, Yasuyoshi Watanabe, Yilong Cui

**Affiliations:** ^1^ Laboratory for Biofunction Dynamics Imaging, RIKEN Center for Biosystems Dynamics Research, Kobe, Japan; ^2^ Department of Chinese Medicine Diagnostics, School of Traditional Chinese Medicine, Beijing University of Chinese Medicine, Beijing, China; ^3^ Institute for Brain Disorders, Dongzhimen Hospital, Beijing University of Chinese Medicine, Beijing, China; ^4^ Laboratory for Labeling Chemistry, RIKEN Center for Biosystems Dynamics Research, Kobe, Japan; ^5^ Laboratory for Pathophysiological and Health Science, RIKEN Center for Biosystems Dynamics Research, Kobe, Japan

**Keywords:** fatigue-like symptoms, regional neuroinflammation, peripheral infection, PET, sickness behavior

## Abstract

**Introduction:**

A series of symptoms, including fever, widespread pain, fatigue, and even ageusia, have frequently been reported in the context of various infections, such as COVID-19. Although the pathogenic mechanisms underlying an infection causing fever and pain have been well established, the mechanisms of fatigue induced by infection in specific brain regions remain unclear.

**Methods:**

To elucidate whether and how the peripheral infection cause fatigue via regional neuroinflammation, we performed a brain-wide investigation of neuroinflammation in a peripheral pseudoinfection rat model using [^18^F]DPA-714 positron emission tomography (PET) imaging analysis, in which the polyriboinosinic: polyribocytidylic acid (poly I:C) was intraperitoneally injected.

**Results:**

Transient fever lasting for several hours and subsequent suppression of spontaneous activity lasting a few days were induced by poly I:C treatment. Significant increase in plasma interleukin (IL)-1β, IL-6 and tumour necrosis factor (TNF)-α were observed at 2 and 4 h following poly I:C treatment. PET imaging analysis revealed that the brain uptake of [^18^F]DPA-714 was significantly increased in several brain regions one day after poly I:C treatment, such as the dorsal raphe (DR), parvicellular part of red nucleus (RPC), A5 and A7 noradrenergic nucleus, compared with the control group. The accumulation of [^18^F]DPA-714 in the DR, RPC and A5 was positively correlated with subsequent fatigue-like behavior, and that in the A7 tended to positively correlate with fever.

**Discussion:**

These findings suggest that peripheral infection may trigger regional neuroinflammation, which may cause specific symptoms such as fatigue. A similar mechanism might be involved in COVID-19.

## 1 Introduction

Fatigue and other prolonged neuropsychiatric and physical manifestations caused by SARS-CoV-2 infection have received growing attention as the most frequently claimed post-COVID-19 sequelae and are becoming a serious global public health issue. In general, infections induced by various kinds of pathogens or pathogenic organisms are known to be associated with a series of symptoms including fever, widespread pain, and fatigue. The underlying mechanisms for infection-evoked fever and pain have been well investigated. In response to peripheral infection, prostaglandin E2 (PGE2) is produced and increases in the brain parenchyma, which activates PGE receptor 3 (EP3) receptors of thermoregulatory neurons in the preoptic area of the hypothalamus leading to fever (1, 2). Peripheral and central mechanisms, such as upregulation of the transient receptor potential family in afferent sensory neurons evoked by pro-inflammatory mediators have been proposed to be involved in infection-related pain (3, 4). Long-term debilitating fatigue and severe fatigue sensations have also been reported frequently in various infections. In 1985, there was an outbreak of illness characterized by chronic or recurrent debilitating fatigue linked to the Epstein–Barr virus in Nevada in USA. The illness was defined as chronic fatigue syndrome (CFS) by the Centers for Disease Control and Prevention and first described in a publication in 1988 (5). Thereafter, similar symptoms have been frequently reported in some virus infections, including coronavirus disease 2019 (COVID-19). The latest clinical studies in COVID-19 have mentioned that besides respiratory symptoms, fatigue is one of the most common (approximately 50%) typical clinical manifestations related to COVID-19, and might be observed as sequelae (6–8). However, the detailed mechanisms pointing to the involvement of infection in fatigue pathophysiology remain unclear, and conclusive evidence has yet to be demonstrated.

Recently, neuroinflammation has been proposed as a possible mechanism related to the development of fatigue. Neuroinflammation, an immune response in the central nervous system (CNS) whereby glial cells are activated, is known to be involved in a variety of CNS diseases. In a pioneering study, Nakatomi et al. (9) reported that widespread neuroinflammation, particularly in the hippocampus, amygdala, thalamus, and midbrain, correlated with the severity of symptoms in patients with CFS. Although peripheral infection has been reported to trigger inflammatory responses in the brain (10–12), the underlying mechanisms for fatigue involved in neuroinflammation in the specific brain regions remain unclear.

The regional neuroinflammation in the brain could be quantitatively evaluated by positron emission tomography (PET) imaging non-invasively using radiolabeled compounds targeting specific biomarkers of activated glial cells. [^18^F]DPA-714 (N,N-Diethyl-2-(2-[4-(2-Fluoroethoxy)-Phenyl]-5,7-Dimethyl-Pyrazolo[1,5-a]Pyrimidin-3-yl)-Acetamide) has been developed and widely used for the quantitative assessment of neuroinflammation in diverse central nervous system diseases as a specific radioligand for the translocator protein 18 kDa, a reliable biomarker for activated microglia (13). To investigate whether and how the regional neuroinflammation is involved in peripheral infection induced fatigue-like symptoms, we induced a peripheral pseudoinfection in rats by intraperitoneal injection of polyriboinosinic: polyribocytidylic acid (poly I:C) (14). Using this animal model, we performed brain-wide quantitative evaluation of neuroinflammation using [^18^F]DPA-714 PET imaging analysis and assessed the correlation between regional neuroinflammation and sickness behaviors, including fatigue.

## 2 Materials and methods

### 2.1 Animals and peripheral pseudoinfection generation

Forty-six male Sprague-Dawley rats (6 weeks old) were purchased from Japan SLC (Hamamatsu, Japan). Since data variability has been reported to be greater in the females than in males in rodents PET imaging study, only male rats was used (15). The rats were housed in a temperature- (23 ± 1°C), humidity- (60 ± 5%), and light- (lights on at 8:00 and off at 20:00) controlled environment. A standard laboratory diet and tap water were available *ad libitum*. For acclimation, rats were housed in the experimental room for at least 1 week before the week-long pre-level measurement of spontaneous activity, and randomly divided into saline- (control) and poly I:C-treated groups. A pseudo-viral infection in rats (8 weeks old) was induced by intraperitoneal injection of poly I:C (GE Healthcare Life Science, Buckinghamshire, UK), a synthetic double-stranded RNA which has been widely used to mimic peripheral viral infections, dissolved in saline at a dose of 10 mg/kg body weight between 10:00 and 11:00 in the morning (14, 16). In the control group, rats were injected with saline at analogous procedure. Body weight was measured in the morning every day from 3 days before to 4 days after the poly I:C injection. The experimental procedures in the present study were approved by the Institutional Animal Care and Use Committee of RIKEN, Kobe Branch, and were performed in accordance with the *Guide for the care and use of laboratory animals* (NIH publication No. 85-23, revised 2011).

### 2.2 Measurement of spontaneous activity

To quantitatively evaluate fatigue state, the spontaneous activity of each rat was recorded with an infrared beam sensor (NS-AS01; Neuroscience, Tokyo, Japan) prior to and following a poly I:C injection. The infrared beam sensor was placed 15 cm above the center of each cage, and the activities of rats housed in individual cages were measured. The level of night-time spontaneous activity was normalized by the mean value of the 3 days prior to poly I:C injection. The fatigue of rats was calculated by assessing night-time spontaneous activity, which was added up every 60 min and analyzed in Clock Lab (Neuroscience, Tokyo, Japan). In addition, the spontaneous activity in all rats used in [^18^F]DPA-714 PET scan was also examined separately throughout the entire experimental period for the correlation analysis.

### 2.3 Body temperature measurement

Body temperature of rats was monitored using an implantable programmable temperature transponder (IPTT-300, Bio Medic Data Systems, Seaford, USA), which was implanted gently into the subcutaneous tissue between the scapulae of each rat under anesthesia (with a mixture of 1.5% isoflurane and nitrous oxide/oxygen 7:3) with a syringe-like action 7 days before intraperitoneal injection of poly I:C or saline. Temporal changes in the body temperature of the rats were measured wirelessly using an IPTT reader from 0 h (before injection) to 48 h following the poly I:C or saline injection.

### 2.4 Cytokine analysis

Besides the pre-injection levels (baseline), at 2 h, 4 h, 8 h, 24 h, and 48 h after poly I:C injection, rats were shortly anesthetized with a mixture of 1.5% isoflurane and nitrous oxide/oxygen (7:3), and blood samples were collected from an indwelling catheter in the tail vein implanted just before each sampling. Venous blood was centrifuged at 12,000 rpm for 10 min at 4°C and cytokine levels were measured on the resulting plasma. The cytokines interleukin (IL)-1β, IL-6 and tumour necrosis factor (TNF)-α were simultaneously assessed using the Bio-Plex Pro Rat Cytokines Assay (Bio-Rad Laboratories, California, USA) (17). Since, the level of plasma cytokines remained stable following repeat measurements in satellite control rats (**Supplemental Figure**), the poly I:C induced temporal changes in plasma cytokines were compared with their own baseline (pre-injection level).

### 2.5 PET scanning

In the present study, [^18^F]DPA-714 was synthesized as reported by Sydney group (18). The product was identified and purified using high-performance liquid chromatography on a COSMOSIL C18-AR-II column (10 × 250 mm, Nacalai, Kyoto, Japan). Molar activity ranged from 33 to 160 GBq/µmol. Radiochemical purity analyzed using HPLC exceeded 99%.

All PET scans were performed using a microPET Focus220 (Siemens, Knoxville, USA) designed for small laboratory animals. Both saline- and poly I:C-treated rats were anesthetized with 1.5% isoflurane and nitrous oxide/oxygen (7:3) and placed in a prone position in the PET scanner gantry. During the PET scan, the body temperature was maintained at 37°C using a small animal warmer connected to a thermometer (BWT-100A; Bio Research Center, Nagoya, Japan). A 45-min emission scan was performed immediately after the bolus injection of [^18^F]DPA-714 (≈75 MBq per animal) via a cannula inserted into the tail vein; the energy window was 400-650 keV and the coincidence time window was 6 ns. Emission data were collected in list mode and sorted into dynamic sonograms (6 × 10 s, 6 × 30 s, 11 × 60 s, and 10 × 180 s, for a total of 33 frames). The acquired data were reconstructed by standard 2D-filtered back projection (FBP) (ramp filter, cutoff frequency at 0.5 cycles per pixel) for quantification, and by a statistical maximum a posteriori probability (MAP) algorithm (12 iterations with point spread function effect) for image registration.

### 2.6 Image analysis

PET images were co-registered to a magnetic resonance imaging (MRI) template which was placed in a Paxinos and Watson stereotactic space using the PMOD imaging processing software (version 3.6, PMOD Technologies, Zürich, Switzerland). Each FBP image was spatially smoothed using an isotropic Gaussian kernel (0.6-mm full width at half maximum) for enhancement of the statistical power. The radioactivity was normalized with cylinder phantom data and expressed as standardized uptake values (SUVs).

A voxel-based statistical analysis was performed using Statistical Parametric Mapping (SPM) 8 software (Welcome Department of Imaging Neuroscience, London, UK). A two sample *t*-test was used for estimating the statistical differences between groups. The statistical threshold was set to be *P* < 0.005 familywise error (FWE) with an extent threshold of 200 contiguous voxels.

### 2.7 Statistical analysis

All results are expressed as the mean ± SEM. All data were analyzed in SPSS (version 24.0, IBM, Armonk, USA). One-way analysis of variance (ANOVA) with Bonferroni’s multiple-comparison procedure was used to assess changes in body temperature, cytokines, and spontaneous activity prior to and following poly I:C injection. Two-way repeated measures ANOVA with Bonferroni’s multiple-comparison procedure was used to assess differences in body temperature and spontaneous activity between the two groups of rats. Pearson’s test was used for correlation analysis of the accumulation of [^18^F]DPA-714 in each brain region and fatigue-like behavior. Differences were considered statistically significant at *P* < 0.05.

## 3 Results

### 3.1 Poly I:C-induced symptoms and plasma cytokine elevation

**Figure 1:**
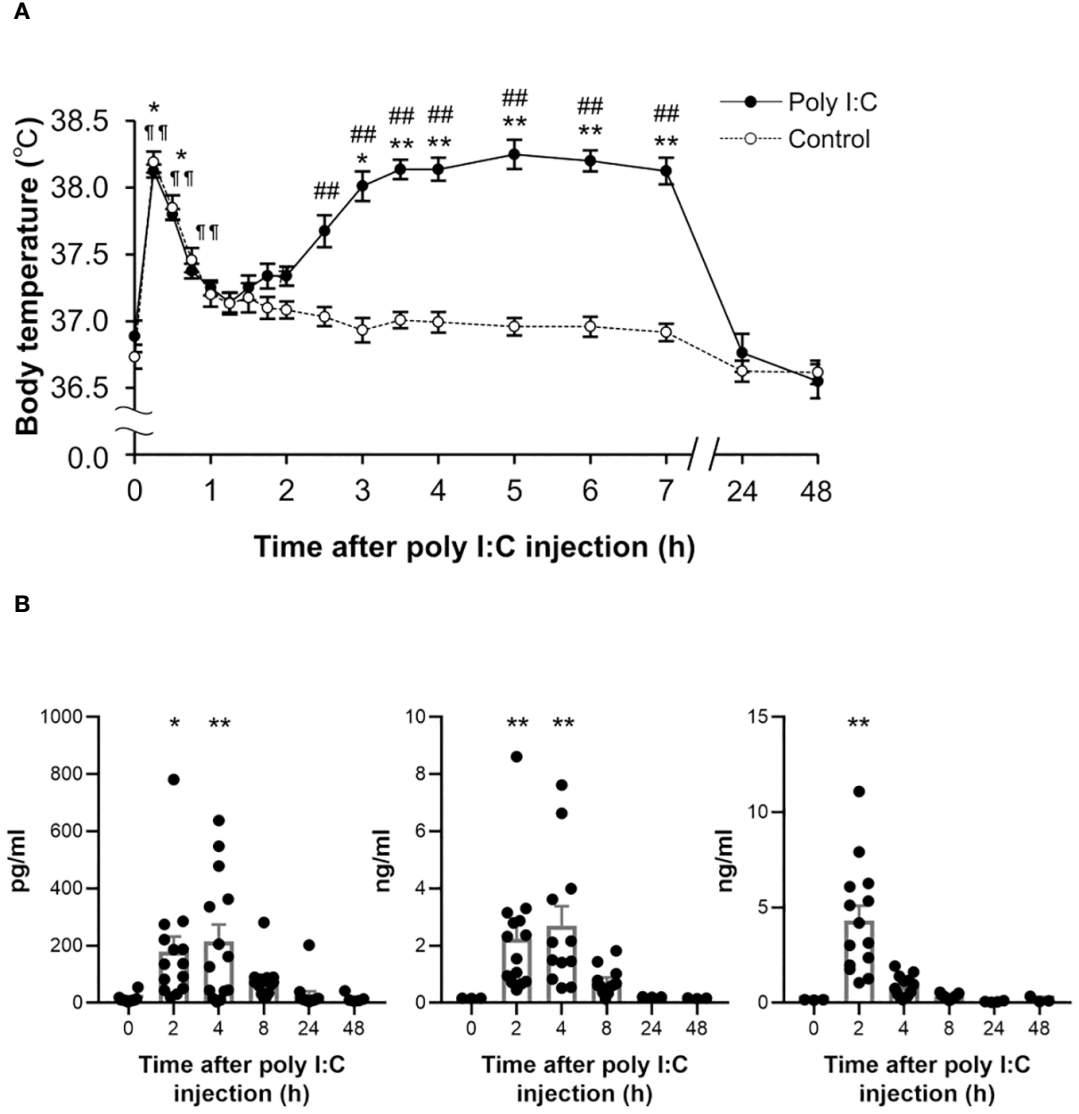
Temporal changes in body temperature and peripheral cytokines following a poly I:C or saline treatment. **(A)** Subcutaneous body temperature of rats from poly I:C (10 mg/kg) treated group (closed circles, *n* = 8) and control group (open circles, *n* = 12) up to 48 h after intraperitoneal injection with poly I:C or saline were plotted. *P < 0.05, **P < 0.01 for poly I:C-treated group, ^¶ ¶^ *P* < 0.01 for control group vs. 0 h (before injection). ^#^ *P* < 0.05, ^##^ *P* < 0.01 vs. control group. **(B)** Plasma IL-1β, IL-6, and TNF-α were detected at 2 h, 4 h, 8 h, 24 h and 48 h following poly I:C injection, as well as pre-injection (0 h). Each value represents the mean ± SEM, *n* = 14. *P < 0.05, **P < 0.01 vs. pre-treated level. IL, interleukin; TNF, tumour necrosis factor.

The body weight of poly I:C-treated rats decreased approximately by 10% of the pre-level value the day after poly I:C injection, thereafter recovering gradually. The body temperature of rats in the poly I:C-treated group increased significantly again and reached a peak at 5 h (*P* < 0.001), following a significant increase as an acute stress response within the first hour after the poly I:C injection (**Figure 1A**).

To assess poly I:C-induced peripheral inflammatory responses, temporal changes in plasma cytokines were detected up to 48 h after the poly I:C injection (**Figure 1B**). Several pro-inflammatory cytokines were significantly elevated at early injection time points, as compared with the pre-level. Two hours after poly I:C injection, cytokines IL-1β (*P* = 0.019), IL-6 (*P* = 0.004) and TNF-α (*P* < 0.001) were significantly elevated. A significant elevation of IL-1β (*P* = 0.002) and IL-6 (*P* = 0.006) was observed until 4 h following the poly I:C injection.

### 3.2 Poly I:C-induced suppression of spontaneous activity

**Figure 2:**
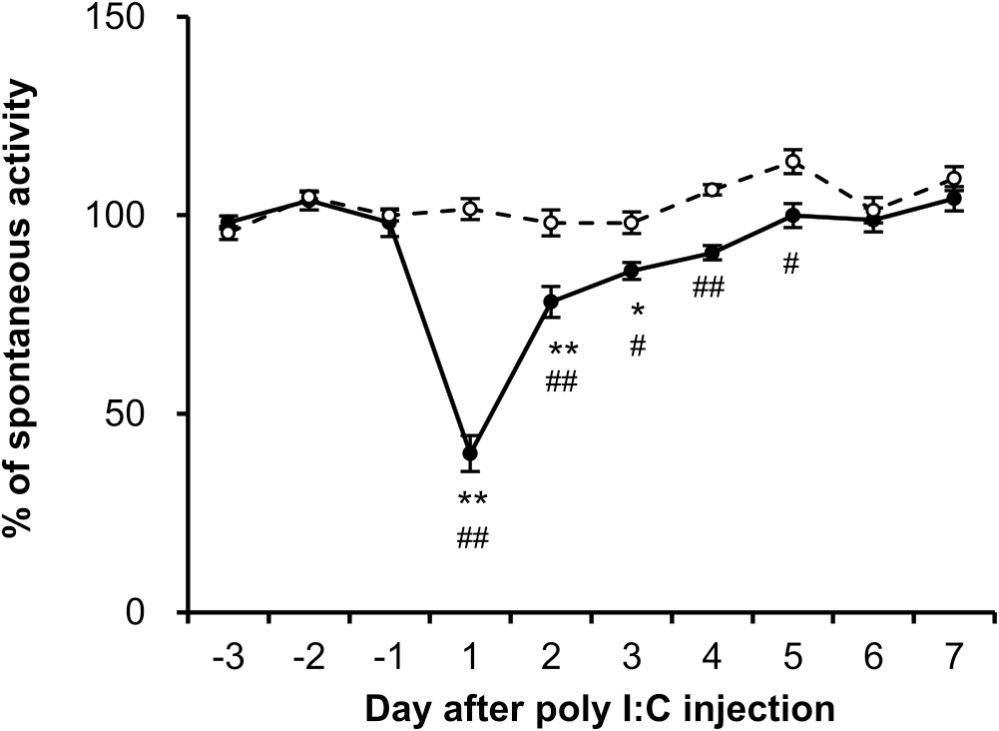
Dynamics of night-time spontaneous activity induced by poly I:C intraperitoneal injection. The spontaneous activity of each rat from control (open circles, *n* = 6) and poly I:C-treated (closed circles, *n* = 6) groups was recorded from 3 days prior to injection, and the percentage of night-time spontaneous activity was normalized by the mean value over the course of the 3 days (-3 to -1). Each value represents the mean ± SEM. *P < 0.05, **P < 0.01 vs. pre-injection level. ^#^ *P* < 0.05, ^##^ *P* < 0.01 vs. control group.

Fatigue can be assessed by changes in voluntary activity, known to be associated with motivation (14). To evaluate fatigue, night-time spontaneous activity in the home cage was investigated in both groups. As shown in **Figure 2**, the night-time spontaneous activity in the control group remained nearly stable throughout the experiment. However, the night-time spontaneous activity decreased significantly on the first night after the poly I:C injection (post day 1, *P* < 0.001). On the second night (post day 2), the night-time spontaneous activity sharply recovered to 78 ± 4% (*P* < 0.001) of its pre-level, and gradually returned to baseline level within 1 week. A significant difference in night-time spontaneous activity between the two groups was observed until day 5 post-injection (*P* < 0.001).

### 3.3 Peripheral infection-induced neuroinflammation

**Figure 3:**
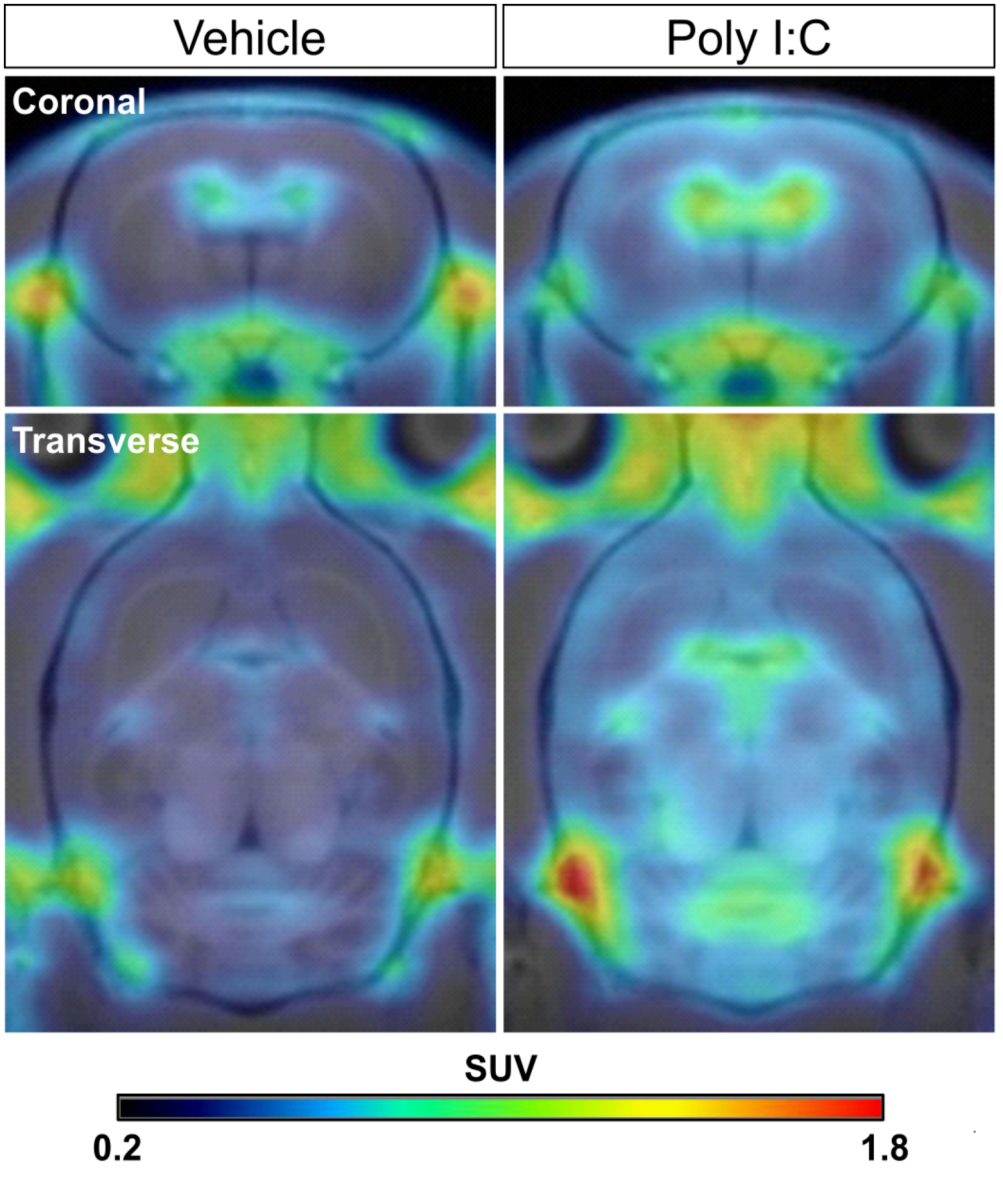
Representative [^18^F]DPA-714 PET images co-registered with an MRI template in the saline- and poly I:C-treated rats. The coronal and transverse views of representative PET images were shown. PET scan with [^18^F]DPA-714 was performed in rats from both groups at 24 h after poly I:C or vehicle (saline) injection. PET images were reconstructed with a MAP algorithm and summed from 5 to 45 min following a [^18^F]DPA-714 bolus injection. MAP, statistical maximum a posteriori probability.

**Figure 4:**
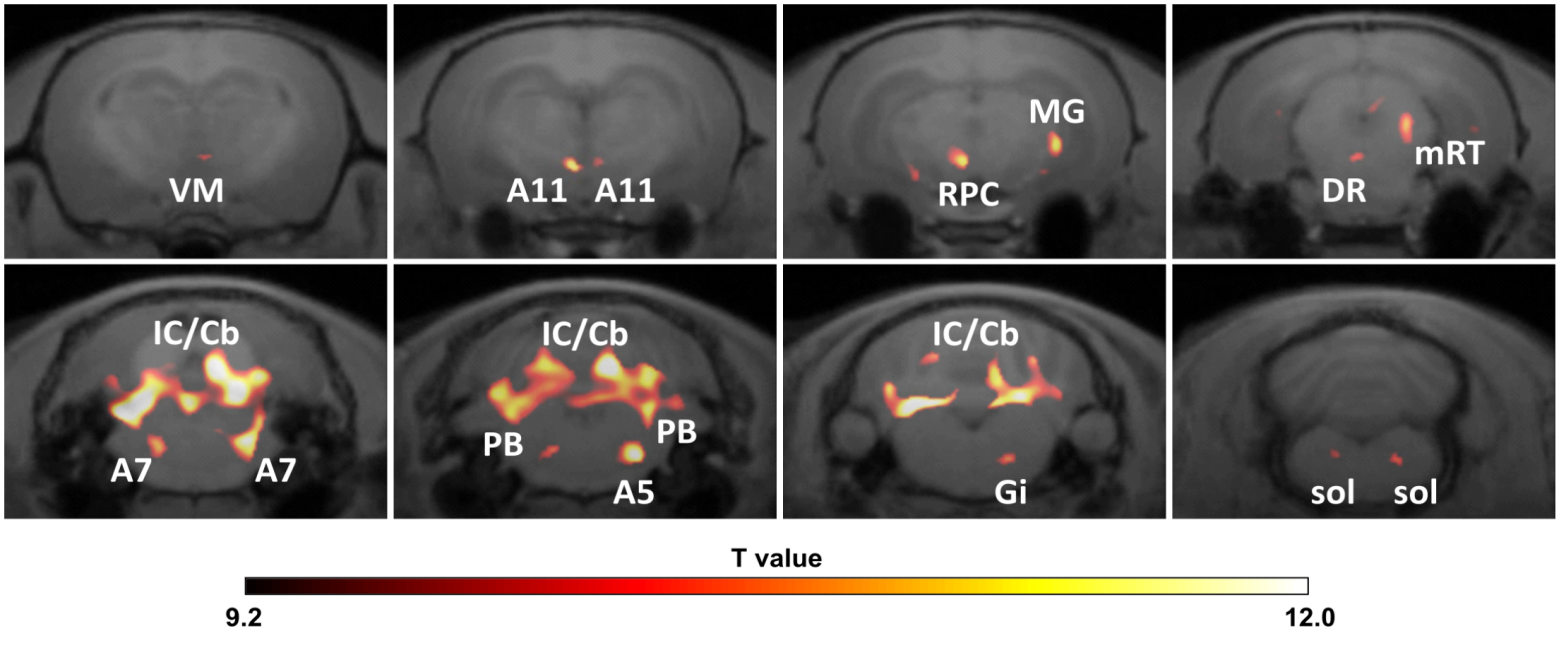
Significant increment of regional neuroinflammation following peripheral pseudoinfection. Images were obtained by voxel-based statistical comparison of [^18^F]DPA-714 accumulation in entire brain regions between vehicle (saline) (*n* = 8) and poly I:C (*n* = 8) injected rats and co-registered with an MRI template. The T value of 9.14 was used as the threshold corresponding to the *P* < 0.005 FWE threshold. The right side of images corresponds to the right hemisphere. A5, A5 noradrenergic nucleus; A7, A7 noradrenergic nucleus; A11, A11 region; DR, dorsal raphe nucleus; Gi, gigantocellular reticular nucleus; IC/Cb, inferior colliculus/cerebellum; mRT, mesencephalic reticular formation; MG, medial geniculate nucleus; PB, parabrachial nucleus; RPC, parvicellular part of red nucleus; sol, nucleus tractus solitarius; VM, ventromedial thalamic nucleus.

In order to confirm whether the peripheral infection would induce neuroinflammation in the brain, a PET scan with [^18^F]DPA-714 was performed in rats from both groups 1 day after the poly I:C or saline injection. As shown in the representative PET images (**Figure 3**), [^18^F]DPA-714 radioactivity was barely observed within the brain in the saline-injected rats, except in the choroid plexus in the cerebral ventricles and some surrounding circumventricular area. However, the radioactivity of [^18^F]DPA-714 apparently increased throughout the brain regions after the poly I:C injection, especially in the mesencephalon and medulla, as well as in the cerebellum. A voxel-based statistical analysis showed that the accumulation of [^18^F]DPA-714 significantly increased in the several brain regions following poly I:C injection, including the dorsal raphe (DR), parvicellular part of red nucleus (RPC), central medial thalamic nucleus (CM), parabrachial nucleus (PB), gigantocellular reticular nucleus (Gi), A5, A7, A11 nuclei, and so on (**Figure 4** and **Table 1**).

**Table 1:** Brain regions of significantly increased [^18^F]DPA-714 accumulation following peripheral pseudo infection.

		T value	Volume
Brain regions	Laterality	(peak)	(mm^3^)
Ventromedial thalamic nucleus, VM	R	9.88	0.16
A11 dopaminergic nucleus, A11	L/R	12.02/10.04	0.5/0.19
Red nucleus, parvicellular part, RPC	L	11.76	0.45
Medial geniculate nucleus, MG	R	11.46	1.39
Mesencephalic reticular formation, mRT	R	10.7	0.68
Dorsal raphe nucleus, DR		10.09	0.17
Dorsolateral periaqueductal gray, DLPAG	R	10.42	0.77
Hippocampus, HC	L	10.5	0.63
Precuneiform area, PrCnF	R	10.8	0.6
Subiculum, transition area, STr	R	10.9	0.37
Entothinal cortex, Ent	R	11.4	0.61
Parasubiculum, PaS	L	10.4	0.34
Cuneiform nucleus, CnF	R	11.31	0.58
Parabrachial nucleus, PB	L/R	12.37/11.4	0.74/0.36
A7 noradrenergic nucleus, A7	L/R	9.83/11.76	0.27/1.25
Pontine reticular nucleus, oral part, PnO	L	9.29	0.96
A5 noradrenergic nucleus, A5	R	12.46	0.98
Gigantocellular reticular nucleus, Gi	R	10.82	0.67
nucleus tractus solitarius, Sol	L/R	9.93/11.56	0.13/0.75
Inferior colliculus/Cerebellum, IC/Cb		13.34	29.58

Vehicle (Saline) (n = 8) versus Poly I:C (10 mg/kg) (n = 8). Height threshold: T = 9.14 with an extent threshold of 200 contiguous voxels, p < 0.005 Familywise Error (FWE) corrected.

### 3.4 Correlation between regional neuroinflammation and fatigue-like behavior

**Figure 5:**
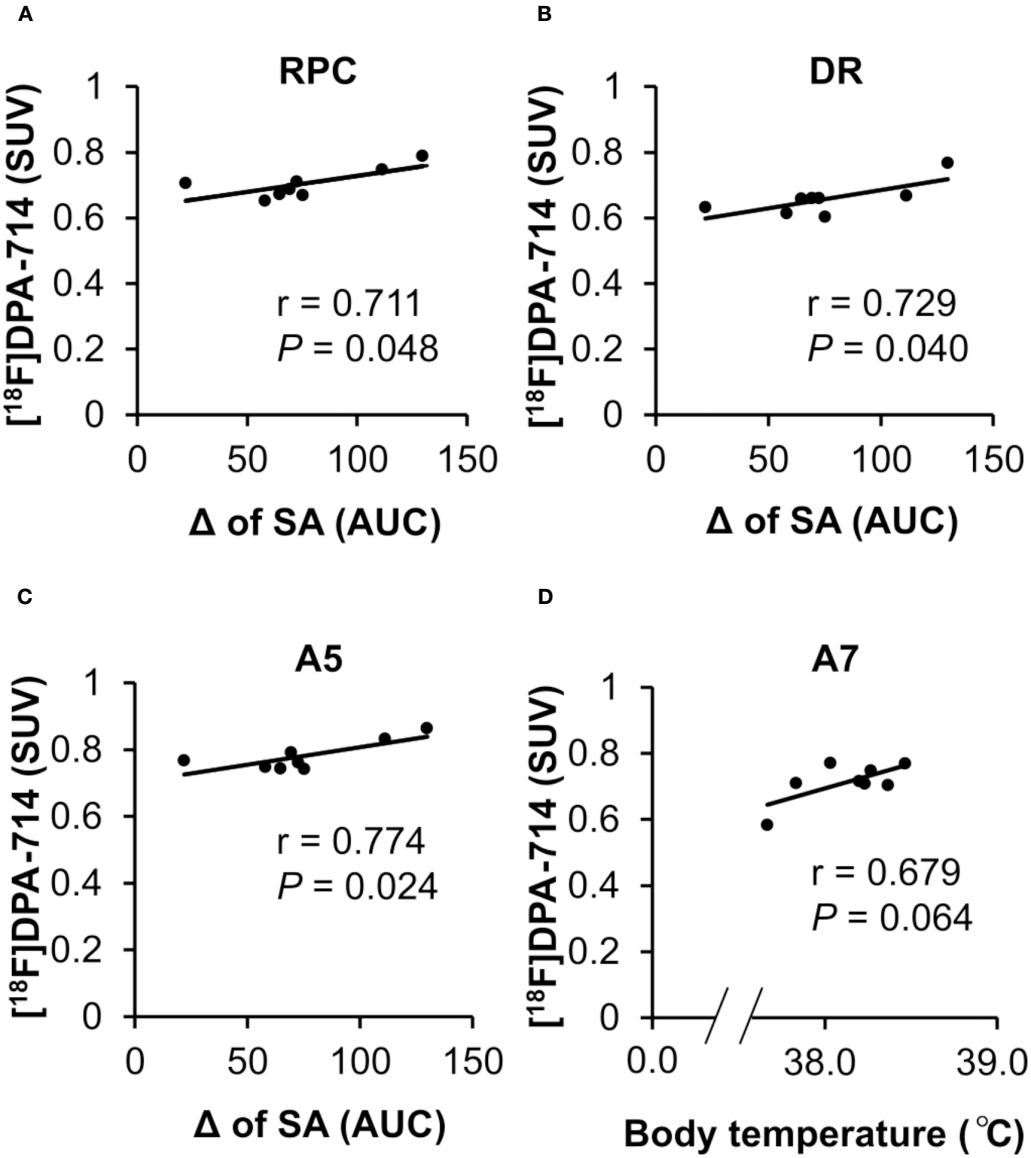
Correlation between regional neuroinflammation in brain areas and fatigue-like behavior. **(A–C)** The correlation between neuroinflammation in the RPC, DR and A5 and night-time spontaneous activity. The positive correlation between the accumulation of [^18^F]DPA-714 in the RPC, DR, and A5 at 24 h after poly I:C injection with a prolonged decrease in night-time spontaneous activity from day 2 to day 5 following poly I:C injection. The Pearson coefficient value (r) is shown for each relation. **(D)** The correlation between neuroinflammation in the A7 and body temperature. The tendency towards a positive correlation between the accumulation of [^18^F]DPA-714 in the A7 at 24 h following poly I:C injection and an elevated body temperature following poly I:C injection. The Pearson coefficient value (r) is shown for the relation. A5, A5 noradrenergic nucleus; A7, A7 noradrenergic nucleus; DR, dorsal raphe nucleus; RPC, parvicellular part of red nucleus; SA, night-time spontaneous activity.

Finally, to assess whether and how those regional neuroinflammations cause peripheral infection-induced symptoms, we analyzed the correlation between the [^18^F]DPA-714 accumulation in all the brain regions showing significant increment and the fever or fatigue-like behavior. The correlation analysis revealed that the [^18^F]DPA-714 accumulation in the DR, RPC and A5 positively correlated with the persistent fatigue severity defined by decrease in spontaneous activity from day 2 to day 5 following the poly I:C injection (**Figures 5A–C**). Moreover, a tendency towards a positive correlation of the [^18^F]DPA-714 accumulation with body temperature was observed in the A7 noradrenergic nucleus (**Figure 5D**).

## 4 Discussion

In this study, we demonstrated that regional neuroinflammation caused by peripheral infection could be involved in fatigue and related symptoms, such as fever. Here, we provide lines of evidence that 1) transient fever and suppressed spontaneous activity lasting a few days were observed after an intraperitoneal injection of poly I:C, which has been widely used for induction of pseudoinfection; 2) an increased accumulation of [^18^F]DPA-714 was found in widespread brain regions 1 day after treatment with poly I:C; 3) a voxel-based statistical analysis showed that a significant increment of [^18^F]DPA-714 accumulation in the brain regions was closely related to fatigue-like behavior. Indeed, the accumulation of [^18^F]DPA-714 in the DR, RPC, and A5, was positively correlated with fatigue severity, and that in the A7 tended to positively correlate with fever. To our knowledge, this is the first brain-wide investigation to determine the region specific neuroinflammation induced by peripheral infection that may relate to fatigue and specific related symptoms.

Pro-inflammatory cytokines, including IL-1β, are known to activate the primary afferent nerve terminal or IL-1 receptors present on perivascular macrophages and endothelial cells, resulting in neuroinflammation following peripheral infection (19–21). An increase in plasma IL-1β concentrations was observed, suggesting that these two signaling pathways may represent pathways for conveying immune signals from the periphery to the brain, in the present study.

The main finding of the present study is that regional neuroinflammation in several brain regions may relate to the pathophysiology of fatigue-like symptoms following peripheral infection, such as the DR, RPC, and A5. Since a PET imaging technique provides a non-invasive approach for the quantitative evaluation of neuroinflammation *in vivo*, the association of regional neuroinflammation with consequent behavioral changes may be observed in the same animal. In the present study, we found that the peripheral infection-induced regional neuroinflammation in the DR was positively correlated with the subsequent fatigue-like symptoms. Functional alternations in the brain serotonergic system have long been implicated in fatigue development and sensation (22). It has been suggested that dysfunction of the serotonergic system could represent an underlying mechanism involved in chronic/pathogenic fatigue (23). In exercise-induced acute/physiological fatigue, the increased biosynthesis and release of serotonin (5-HT) in several brain regions have been reported to be involved in fatigue sensations (23, 24). In contrast, selective serotonin reuptake inhibitors, which result in an increase in extracellular serotonin concentration, have been demonstrated to be effective for some patients with CFS. A gene polymorphism analysis in CFS patients by our group demonstrated that the frequency of longer (L or XL) allelic variants of the 5-HT transporter (5-HTT) promoter region was significantly increased compared to that in controls, pointing to elevated 5-HTT expression and low levels of extracellular 5-HT concentrations in CFS patients (25). Moreover, clinical studies have also demonstrated that the upregulation of 5-HTT and consequent reduction of extracellular 5-HT levels were observed in IFN-α and IFN-γ therapies to treat various forms of cancer and hepatitis C, in which patients often complain of serious tiredness (26, 27). These observations suggest that the dysfunction of serotonergic system could represent an underlying mechanism involved at least in chronic/pathogenic fatigue. Along with the fact that neuroinflammation is known to induce dysfunction or decline in regional neural activity (28), the results in the present study suggest that regional neuroinflammation in the DR probably cause fatigue-like behavior via functional changes in the serotonergic system. In addition, neuroinflammation in the RPC and the A5 noradrenergic nucleus were also positively associated with fatigue-like behavior. Recently, a positive correlation has been reported between the magnitude of atrophy in the superior cerebellar peduncle (Scp) which envelops and traverses the RPC at all rostrocaudal leves, and fatigue severity in multiple sclerosis patients (29), and such volumetric variation in the Scp was then considered as an early structural change preceding fatigue development (30). Overall, these observations suggest that regional neuroinflammation in these brain areas could be a plausible mechanism underlying peripheral infection-induced fatigue-like symptoms. Incidentally, chronic fatigue has been reported to be one of most frequently reported symptoms following COVID-19 infection, in which the elevation of IL1-family cytokines was also observed (7, 31), suggesting that a similar mechanism underlying neuroinflammation in multiple brain regions might be involved in such fatigue evoked by COVID-19.

In the present study, we also found that neuroinflammation in several other brain regions, including the A7, A11, CM, PB, and Gi, was significantly increased, but was not correlated with fatigue-like behavior. The tendency towards a positive association between [^18^F]DPA-714 accumulation in the A7 and fever was observed following poly I:C treatment. Peripheral infection-induced PGE2 in A7 has been reported to suppress the inhibitory innervation of the A7 noradrenergic nucleus to the rostral medullary raphe (RMR), resulting in fever (32). In the present study, although the correlation was weak owing to the mismatched [^18^F]DPA-714 PET scan timing, it indicated that neuroinflammation in A7 may be implicated in fever (**Figure 1A**). Taken together, these results suggested that the peripheral infection-induced diverse symptoms were probably attributed to regional neuroinflammation in specific brain areas.

In conclusion, in the present study, we performed a brain-wide investigation to provide prospective evidence of the brain regions of peripheral infection-induced neuroinflammation. We also demonstrated the effect of regional neuroinflammation to fatigue and specific related symptoms. Future research is needed to further clarify the multiple interactions of these symptoms, which will aid in the development of more effective treatment strategies based on anti-inflammatory effects to address all fatigue related symptoms.

## Data availability statement

The original contributions presented in the study are included in the article/**Supplementary Material**. Further inquiries can be directed to the corresponding author.

## Ethics statement

The animal study was approved by Institutional Animal Care and Use Committee of RIKEN, Kobe Branch. The study was conducted in accordance with the local legislation and institutional requirements.

## Author contributions

DL: Formal Analysis, Investigation, Writing – original draft, Writing – review & editing. DH: Formal Analysis, Investigation, Writing – original draft, Writing – review & editing. YO: Formal Analysis, Investigation, Writing – original draft, Writing – review & editing. WA: Methodology, Resources, Writing – original draft. AM: Methodology, Resources, Writing – original draft. MS: Investigation, Writing – original draft. YWu: Investigation, Writing – original draft. EH: Investigation, Writing – original draft. HN: Investigation, Writing – original draft. TT: Investigation, Writing – original draft. YWad: Data curation, Methodology, Writing – original draft. FL: Formal Analysis, Writing – review & editing. HD: Methodology, Resources, Writing – original draft. YWat: Formal Analysis, Supervision, Writing – review & editing. YC: Conceptualization, Formal Analysis, Investigation, Supervision, Writing – original draft, Writing – review & editing.
