# Supplementary material for: Regional neuroinflammation induced by peripheral infection contributes to fatigue-like symptoms: a [18F]DPA-714 positron emission tomography study in rats
Source: Front Immunol. 2023 Nov 9;14:1261256. doi: 10.3389/fimmu.2023.1261256 (PMC10665845; doi:10.3389/fimmu.2023.1261256)
Supplement: Supplementary file 1 [file DataSheet_1.pdf]

Supplemental Figure

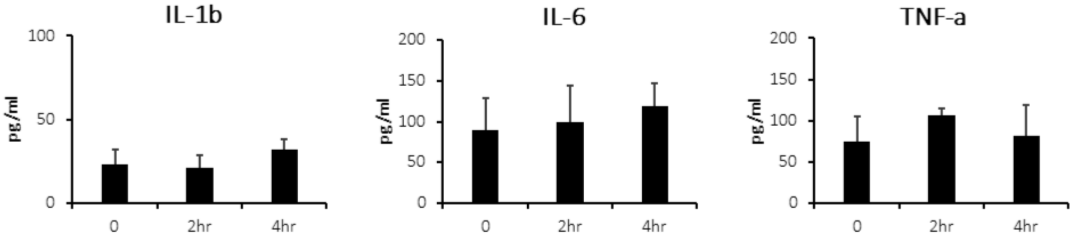

Plasma IL-1β, IL-6, and TNF-α were detected at 2 h, and 4 h following saline injection, as well as pre-injection (0 h) in control rats. Each value represents the mean ± SEM, *n* = 3. IL, interleukin; TNF, tumour necrosis factor.
